# Health-related quality of life in Her2-positive early breast cancer woman using trastuzumab: A systematic review and meta-analysis

**DOI:** 10.3389/fphar.2023.1090326

**Published:** 2023-04-14

**Authors:** Sudewi Mukaromah Khoirunnisa, Fithria Dyah Ayu Suryanegara, Didik Setiawan, Maarten Jacobus Postma

**Affiliations:** ^1^ Department of Health Sciences, University of Groningen, University Medical Center Groningen, Groningen, Netherlands; ^2^ Department of Pharmacy, Institut Teknologi Sumatera, Lampung Selatan, Indonesia; ^3^ Department of Pharmacy, Universitas Islam Indonesia, Yogyakarta, Indonesia; ^4^ Faculty of Pharmacy, Universitas Muhammadiyah Purwokerto, Banyumas, Indonesia; ^5^ Center for Health Economic Studies, Universitas Muhammadiyah Purwokerto, Banyumas, Indonesia; ^6^ Department of Economics, Econometrics and Finance, University of Groningen, Groningen, Netherlands; ^7^ Department of Pharmacology and Therapy, Faculty of Medicine, Universitas Airlangga, Surabaya, Indonesia; ^8^ Centre of Excellence in Higher Education for Pharmaceutical Care Innovation, Universitas Padjadjaran, Bandung, Indonesia

**Keywords:** trastuzumab, breast cancer, meta-analysis, health-related quality of life, Her2-positive breast cancer

## Abstract

**Background:** Despite the benefits of trastuzumab in many trials, evidence of its impact on health-related quality of life (HRQoL) in early treatment has not been summarized. This study explored the effects of trastuzumab treatment on HRQoL, including pooled meta-analysis, in an effort to provide an integrated assessment of HRQoL for Her2-positive early breast cancer patients.

**Methods:** A comprehensive literature review to February 2023 using three databases, focusing on treatment using trastuzumab during the early stage, was performed. The mean changes from baseline during and after treatment were extracted from the included randomized control trials (RCTs) papers and total HRQoL scores were obtained from cross-sectional studies included. Mean difference (MD) and 95% confidence intervals were assessed by a random effect or fixed effect model based on heterogeneity (I^2^).

**Results:** A total of ten studies were identified and reviewed, consisting of seven RCTs and three cross-sectional studies. The pooled analysis of the mean change from baseline during treatment resulted in an MD of 1.92 (95% CI = 1.59 to 2.25, *p* < 0.05, I^2^ = 0%), favoring the trastuzumab group. A non-significant result of the mean change from baseline after treatment appeared in the analysis of 12-month follow-up. In the cross-sectional studies, pooled analyses of HRQoL showed that trastuzumab meaningfully demonstrated an improved HRQoL profile (MD = 9.29, 95% CI = 1.31 to 17.27, *p* = 0.02, I^2^ = 0%).

**Conclusion:** Trastuzumab as a targeted therapy resulted in a favorable effect on HRQoL in the early stages of Her2-positive breast cancer. The findings of significant improvements in patients’ HRQoL and less clinically meaningful deterioration in side effects of trastuzumab-containing regimen during treatment were supported by prolonged survival.

## 1 Introduction

In 2020, the most diagnosed form of cancer in women was breast cancer, with almost 2.4 million incidents and 684.926 mortalities worldwide (Bray et al., 2018; Sung et al., 2021). Her2-positive breast cancer constituted approximately 20% of this total number (Slamon et al., 1989; Wolff et al., 2013). This subtype is typically characterized by an overexpression of the epidermal growth factor receptor 2 (Her2 or erbB2) and rapid growth, both recurring and metastatic (Ross et al., 2009; Goddard et al., 2011; Rimawi et al., 2015). In particular, the poor prognosis of Her2-overexpressed patients is associated with lower disease-free survival and overall survival (Piccart et al., 2001). Likewise, the progression of Her2-positive breast cancer severely decreases the survivors health-related quality of life (HRQoL) due to the detrimental effects of the disease. Patients experience irreversible physical and psychosocial syndromes, mental distress, and symptoms as a tresult of long-term treatment and disease progression (Perry et al., 2007; Bouskill and Kramer, 2016; Hamer et al., 2016; Park et al., 2019). HRQoL has been taken into account in the consideration as a relevant clinical outcome of the treatment strategies for advanced disease (Goodwin et al., 2003; Lemieux et al., 2011; Meldahl et al., 2013). Yet, analyses of trastuzumab treatment in early disease are still scarce.

Trastuzumab, a targeted therapy, is remarkably effective in treating the Her2 overexpression of breast cancer (Piccart-Gebhart et al., 2005; Romond et al., 2012; Goldhirsch et al., 2013; Perez et al., 2014). Trastuzumab has proven to be effective in improving the clinical outcome of the management of the early stages of Her2-positive breast cancer, including disease-free survival, overall survival, and invasive disease-free survival (Perez et al., 2014; Cameron et al., 2017; Piccart et al., 2021). Furthermore, trastuzumab combined with chemotherapy has been used as standard of care in the adjuvant setting and is a preferable choice in high-risk node-negative Her2-positive cases (von Minckwitz et al., 2017). Many studies (Adamowicz and Baczkowska-Waliszewska, 2020; Oh et al., 2021; Dal Lago et al., 2022) evaluating the HRQoL of breast cancer patients treated with monoclonal antibody showed a beneficial effect compared to those who did not receive the treatment.

Despite the benefits of trastuzumab in many trials (Gonzalez-Angulo et al., 2006; Viani et al., 2007; Bria et al., 2008; Dahabreh et al., 2008; Madarnas et al., 2008; Ward et al., 2009; Yin et al., 2011; Mates et al., 2015; O’Sullivan et al., 2015; Long et al., 2016; Genuino et al., 2019), evidence of its impact on HRQoL in early treatment has not been summarized. In particular, this study explored the effects of trastuzumab treatment on HRQoL, including pooled meta-analysis in an effort to provide an integrated assessment of HRQoL for Her2-positive early breast cancer patients.

## 2 Methods

### 2.1 Review strategy

Three electronic databases, PubMed, Embase, and Scopus, were used to conduct a comprehensive review of articles published until February 2023. The search terms included the domains of a) breast cancer, b) trastuzumab, c) quality of life, and d) quality-adjusted life years. A snowball search for additional potential articles found in the reference list of the main articles was subsequently carried out manually to prevent unretrieved papers not being discovered. The search terms and strategies are shown in the Supplementary material **.**

### 2.2 Eligibility criteria

The articles were included according to the following criteria: 1) studies included a female population above 18 years old with Her2-positive breast cancer in early stage who received trastuzumab in any cancer treatment regimen; 2) studies reported HRQoL data, using validated instruments; 3) studies were designed as both randomized controlled trials (RCTs) and observational studies; 4) studies presented the mean change in HRQoL from baseline for RCTs; 5) studies presented the mean HRQoL for cross-sectional studies. The following articles were excluded from this review: systematic reviews, opinion pieces, protocols, commentaries, studies presenting outcomes other than HRQoL studies providing only an abstract, posters and oral presentations. Year and language restrictions were not applied in this systematic review. Duplicates or the same data from multiple publications were only used once.

### 2.3 Study selection

Two reviewers (SMK and FDAS) independently assessed potential articles, including titles and abstracts, based on the eligibility criteria mentioned above. Studies fitting the inclusion criteria were retrieved and assessed if the full-text was available. Full-text articles were then evaluated for final inclusion. Any disparities between the two assessors were solved through discussion until consensus was achieved. The justifications for the eligibility of each study were recorded and presented in a Preferred Reporting Items for Systematic Reviews and Meta-Analyses (PRISMA) flow diagram.

### 2.4 Data extraction

The data from the selected articles were copied to a Microsoft Excel^®^ spreadsheet by SMK and double-checked by FDAS. The details of the data included were: author, date of publication, study design, study period, country, age range, stage of breast cancer, type of instrument, interventions, outcome, and conclusion. Outcomes are presented as changes in HRQoL measurements from baseline to specified moments of follow-up and/or the mean of HRQoL.

### 2.5 Quality evaluation

Two authors (SMK and FDAS) performed the quality evaluations and solved disagreements through consensus. Methodological quality evaluation was assessed using the Cochrane Risk of Bias tool (RoB 2.0) for randomized studies and Risk of Bias in Non-randomised Studies of Interventions (ROBINS-I) for cross-sectional studies. RoB 2.0 consists of five main domains which provide extensive points of analysis, including deviations of post-randomization for intervention. The new version of this tool also enables more up-to-date explanations regarding the measurement of the trials treatment effect and explicitly concerns the particular outcome estimations. In addition, the tool makes use of a series of signaling questions, prompting a reasonable response and simple answers and providing an algorithm to summarize the outcome of each question (Sterne et al., n.d.; Hernán and Robins, 2017). ROBINS-I consists of seven bias domains and signaling questions to inform judgments of the risk of bias and provides a structure approach in evaluating non-randomized studies of interventions (Sterne et al., 2016).

For reporting the evidence quality of the review, the grading of recommendation, assessment, development and evaluation (GRADE) was employed. It offers a systematic method for formulating clinical practice recommendations and is a straightforward framework for creating and presenting evidence summaries (Guyatt et al., 2008; Meader et al., 2014).

### 2.6 Data synthesis and analysis

Forinclusion into the subsequent meta-analysis, the studies should provide the mean change from baseline and/or the mean of HRQoL including the standard deviation (SD) as well as explicitly comparing trastuzumab-containing regimen versus treatment regimen without trastuzumab. The quantitative analysis was done separately for the RCTs and the observational studies. The interpretation of the pooled analysis was determined by the heterogeneity, quantified by Cochran’s Q and the heterogeneity index I^2^. A random-effects model was used if the heterogeneity (I^2^) was high. A result of *p* < 0.05 in Cochran’s Q test was considered to indicate that the variability of the forest plot was significant (Higgins and Thompson, 2002).

## 3 Results

### 3.1 Selection criteria

A total of 6,315 articles were extracted during the initial electronic databases search. 2,720 articles remained after removing duplicates. These articles were then filtered by title, abstract, and text, yielding 261 full-text articles. After retrieving the full texts, 172 articles appeared to be only abstract, leaving 89 articles to review for eligibility.

**FIGURE 1:**
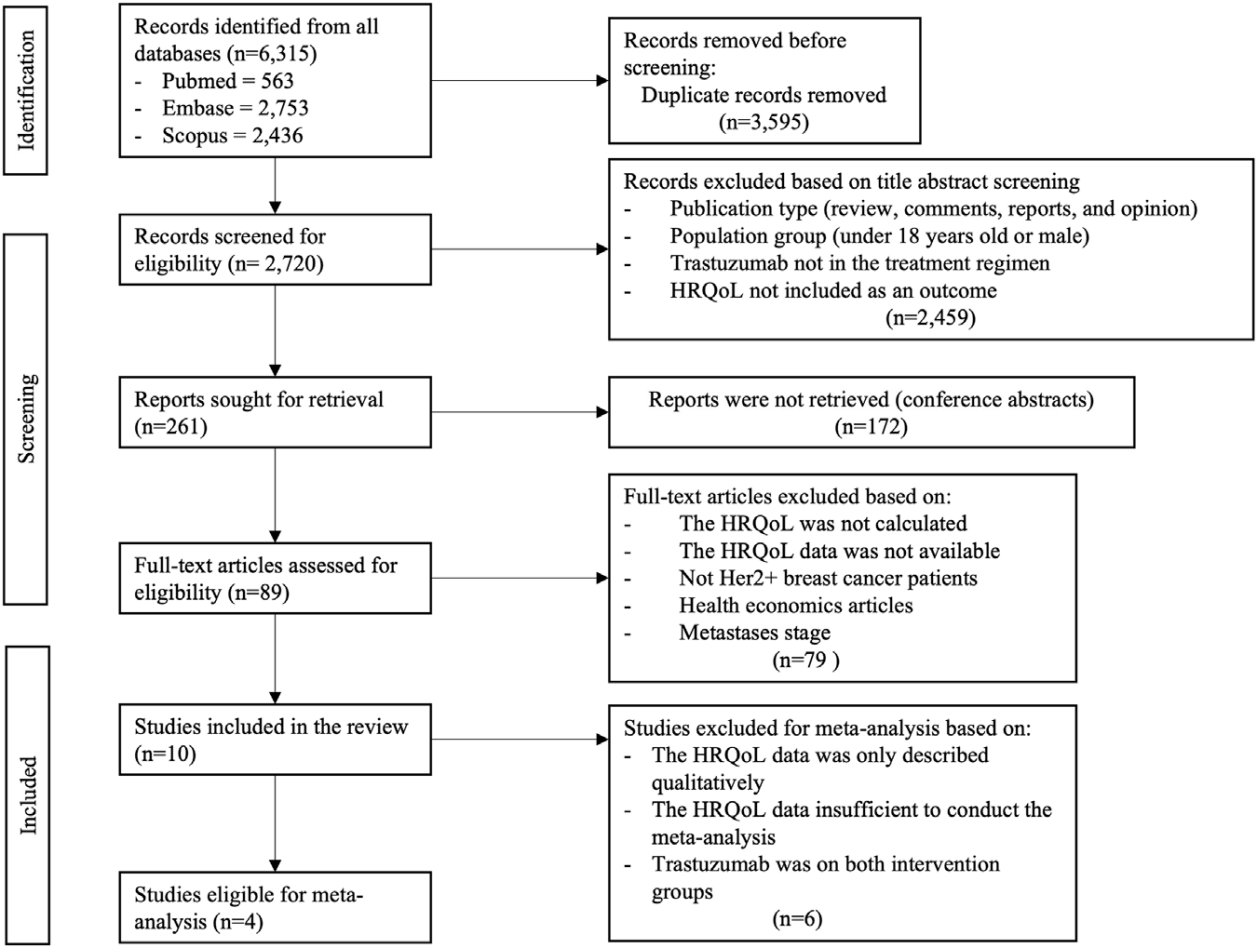
Prisma flow diagram.

Eighty-one articles were eliminated according to stricter application of the inclusion criteria, leaving ten studies for our present analysis, with four of these studies being eligible for meta-analysis (Au et al., 2013; Syrios et al., 2018; Trinca et al., 2019; Conte et al., 2020). Six articles were excluded from quantitative meta-analysis due to lack of detailed data for allowing meta-analysis (Earl et al., 2020; Sawaki et al., 2020, 2022; Sella et al., 2022) or comparing trastuzumab in both arms (Bines et al., 2021; Taira et al., 2021). The details of the selection process are described in Figure 1.

### 3.2 Characteristics of the included studies

The included studies consisted of seven RCTs (Au et al., 2013; Conte et al., 2020; Earl et al., 2020; Sawaki et al., 2020; Bines et al., 2021; Taira et al., 2021; Sella et al., 2022) and three observational studies of cross-sectional design (Syrios et al., 2018; Trinca et al., 2019; Sawaki et al., 2022). Two studies (Trinca et al., 2019; Conte et al., 2020) involved the comparison of a group receiving trastuzumab treatment with a group receiving chemotherapy. Two studies (Au et al., 2013; Syrios et al., 2018) included more than three comparisons of interventions. Three studies (Sawaki et al., 2020, 2022; Taira et al., 2021) investigated the use of trastuzumab monotherapy compared to a combination of trastuzumab and chemotherapy in the treatment of elderly breast cancer patients. Two studies (Conte et al., 2020; Sella et al., 2022) compared trastuzumab regimens with trastuzumab emtansine (T-DM1) was utilized in a study. In addition, one study (Earl et al., 2020) presented a comparison of the HRQoL profile after 6 months of trastuzumab with the HRQoL profile after 12 months of trastuzumab and a study (Bines et al., 2021) evaluated the addition of pertuzumab in trastuzumab in HER-2 positive early breast cancer. Characteristics of the included studies are shown in Table 1.

**TABLE 1:** Characteristics of the included studies.

No	Author, year	Study design	Type of instrument for HRQoL	Follow-up/study period	Data collection	Instrument completion rate, %
**1**	Au et al. (2013)	Randomized controlled trial	EORTC-QLQ-C30, EORTC-QLQ-BR23	Follow-up after 12 months	Self-administered	72%–93%
**2**	Conte et al. (2020)	Randomized controlled trial	EORTC-QLQ-C30, EORTC-QLQ-BR23	Follow-up after12 months 12 months	Self-administered	85%
**3**	Trinca et al. (2019)	Cross-sectional	EORTC-QLQ-C30	Study period was April to November 2016	No reported	Not reported
**4**	Sawaki et al. (2020)	Randomized controlled trial	FACT-G	Study period was 3 years	Self-administered	86%
**5**	Syrios et al. (2018)	Cross-sectional	EORTC-QLQ-C30, EORTC-QLQ-BR23	Study period was December 2015 to May 2016	Self-administered	Not reported
**6**	Taira et al. (2021)	Randomized controlled trial	FACT-G	Follow-up after 2, 12, and 36 months	Self-administered	77%–86%
**7**	Earl et al. (2020)	Randomized controlled trial	EQ-5D-3L	Follow-up after 24 months	Self-administered	80%
**8**	Bines et al. (2021)	Randomized controlled trial	EORTC-QLQ-C30, EORTC-QLQ-BR23	Follow-up after 18, 24, and 36 months	Self-administered	87%–97%
**9**	Sella et al. (2022)	Randomized controlled trial	FACT-B	Study period: 18 months	Self-administered	72%
**10**	Sawaki et al. (2022)	Cross-sectional	FACT-G	Follow-up after 36 months	Self-administered	50%–56%

**Abbreviations:** EORTC-QLQ-C30, European Organization for the Research and Treatment of Cancer Quality of Life Questionnaire; EORTC-QLQ-BR23, The European Organization for Research and Treatment of Cancer Quality of Life Questionnaires-Breast 23; FACT-G, The Functional Assessment of Cancer Therapy—General; EQ-5D-3L: EuroQol-5, dimensions-5 levels.

### 3.3 Data collection method

Health-related quality of life was calculated using valid instruments in all the selected studies. The majority of the studies (Au et al., 2013; Syrios et al., 2018; Trinca et al., 2019; Conte et al., 2020; Bines et al., 2021) made use of the EORTC-QLQ-C30 questionnaire to estimate the HRQoL of women suffering from Her2-positive breast cancer, with three (Syrios et al., 2018; Conte et al., 2020; Bines et al., 2021) of these studies also making use of EORTC-QLQ-BR23. Other types of questionnaires identified were FACT-G (Sawaki et al., 2020, 2022; Taira et al., 2021), FACT-B (Sella et al., 2022), and EQ-5D-3L (Earl et al., 2020). Most of the instruments of the studies included was self-administered with the completion rate ranged from 50% to 97% (Table 1).

### 3.4 Timeline of observations

One trial (Conte et al., 2020) described the mean change of HRQoL during cycles 5 and 11, at the end of chemotherapy, after 6 months of follow-up and after 1 year while another (Au et al., 2013) described the mean change during cycle 4, at the end of chemotherapy, and after 12 months of follow-up. One study (Sawaki et al., 2020) presented observations made after 2 months and 1 year and two studies (Taira et al., 2021; Sawaki et al., 2022) observed the HRQoL after 36 months. The HRQoL of early breast cancer patients filled the questionnaire at 0, 3, 6, 9, 12, 18, and 24 months of follow-up (Earl et al., 2020). Patients in a study (Bines et al., 2021) completed a questionnaire on weeks 10, 13, 25, and at the end of HER2-targeteted therapy (12 months) while another study on weeks 18 (Sella et al., 2022). Finally, an HRQoL profile was made of patients receiving treatment during the observational studies (Syrios et al., 2018; Trinca et al., 2019).

### 3.5 Characteristics of patients

The patients characteristics, interventions used, and qualitative summary of the HRQoL profile was presented in Tables 2, 3. Two studies (Conte et al., 2020; Earl et al., 2020) recruited patients who had previously been treated with anthracycline, taxane, and trastuzumab in the neo or adjuvant setting. One study (Trinca et al., 2019) observed female patients who had already undergone a breast cancer regimen 2 months before the study was conducted and excluded cerebral metastasis patients. Elderly patients in the early stages of breast cancer who underwent curative surgery but who had not been previously treated with chemotherapy or endocrine therapy, had Eastern Coopertive Oncology Group (ECOG) performance status score 0 or 1, and had sufficient organ function were involved in three studies (Sawaki et al., 2020, 2022; Taira et al., 2021). A study allowed patients who has been received surgery for the treatment within 90 days (Sella et al., 2022). One specific study allowed for concurrent treatment with pertuzumab (Syrios et al., 2018). Finally, one trial (Au et al., 2013) involved women aged 18 to 70 with HER2-positive early breast cancer who had undergone either mastectomy or lumpectomy and had Karnofsky performance status ≥80%.

**TABLE 2:** Profile of HRQoL for RCTs.

Author, year	Population	Intervention	Comparison	Outcome		Comments
				Mean change from baseline during treatment	Mean change from baseline after treatment	
Au et al. (2013)	Patients ≥18 years and ≤70 years old- Patients had Karnofsky performance status ≥80%	Doxorubicin 60 mg/m2 and cyclophosphamide 600 mg/m2 for four cycles followed by docetaxel 100 mg/m2 for four cycles and trastuzumab 4 mg/kg loading dose and 2 mg/kg weekly for one year; n baseline = 920, n follow-up = 687	Doxorubicin 60 mg/m2 and cyclophosphamide 600 mg/m2 for four cycles followed by docetaxel 100 mg/m2 for four cycles; n baseline = 841, n follow-up = 610	Doxorubicin, cyclophosphamide, docetaxel, trastuzumab -5.2 (2.868)Doxorubicin, cyclophosphamide, docetaxel -7.1 (3.916)	Doxorubicin, cyclophosphamide, docetaxel, trastuzumab 2.8 (1.544) Doxorubicin, cyclophosphamide, docetaxel 3.7 (2.041)	HRQOL: the deterioration from the baseline was not significant. Show improvement after 12 months. Physical functioning: trastuzumab group exhibited a significant improvement from baseline in the mid-point observations Systemic side effects: significant decrease from baseline for all intervention groups at mid point. After 12 months, an improvement was shown, HRQoL recovered in all domains
Conte et al. (2020)	Patients previously treated by taxane-based chemotherapy or anthracyclines and alkylating agents	Trastuzumab 6 mg/kg IV every three weeks and adjuvant endocrine; n baseline = 632, n follow-up = 384	Trastuzumab emtansine (T-DM1) 3.6 mg/kg IV every three weeks and adjuvant endocrine; n baseline = 655, n follow-up = 430	Trastuzumab, adjuvant endocrine 0.6 (14.052)T-DM1, adjuvant endocrine -1.9 (14.257)	Trastuzumab, adjuvant endocrine 3.2 (15.376)T-DM1, adjuvant endocrine 2.8 (15.618)	HRQoL and physical function: maintenance for both groups until 12 months of follow-upThe mean change from baseline did exceed the threshold in the T-DM1 groupA significant deterioration shown in the T-DM1 group in terms of fatigue, nausea, appetite loss, constipation, pain, systematic therapy side effects domainsMean scores generally returned to the baseline levels after withdrawing treatment
Sawaki et al. (2020)	Patients 70-80 years old Patients with Eastern Coopertive Oncology Group (ECOG) performance status score 0 or 1Patients had sufficient organ function Patients who have not received chemotherapy, prior hormonal treatment was allowed	Trastuzumab 8 mg/kg and a maintenance dose of 6 mg/kg every three weeks for one year; n baseline = 135, n follow-up = 116	Trastuzumab with chemotherapy; nbaseline=131, nfollowup=115	Not reported	Clinically meaningful HRQoL improvement rate at two months (38% for trastuzumab monotherapy vs 15% for trastuzumab plus chemotherapy; P = 0.01), and at one year (43% vs 25%; P = 0.021). There was no significant difference between the two groups at three years	Significant improvement of HRQoL in trastuzumab monotherapy group at two months and one year
Taira, et al. (2021)	Patients 70-80 years old Patients with ECOG performance status score 0 or 1 Patients had received curative surgeryPatients who have not received chemotherapy, prior hormonal treatment was allowed	Trastuzumab monotherapy; n = 137	Trastuzumab and chemotherapy; n=138	Trastuzumab monotherapy 80.4 (15.0) Trastuzumab with chemotherapy 74.5 (15.9)	Trastuzumab monotherapy 79.1 (17.0) Trastuzumab with chemotherapy 78.5 (16.9)	Trastuzumab monotherapy led to a significant improvement in HRQoL Trastuzumab plus chemotherapy led to a meaningful deterioration of HRQoL in the first 36 months of follow-upAfter 36 months, a deterioration was not shown in both groups
Earl, et al. (2019)	Patients ≥18 years old Patients had an indication to be treated by chemotherapy before iniating trastuzumab	6 months trastuzumab (nine cycles); n = 2,043	12 months Trastuzumab (18 cycles); n=2,045	Not reported	HRQoL is shown to decrease during the first three months in both groups	A comparison of general health profiles in both intervention groups
Bines et al. (2021)	Patients had adequate baseline organ function (hematologic, hepatic, and renal), and no active or history of cardiac disease were included	Pertuzumab 840 mg loading dose, followed by 420 mg IV 3-weekly, trastuzumab 8 mg/kg loading dose, followed by the maintenance dose of 6 mg/kg IV every 3 weeks, chemotherapy (anthracycline based or not); n = 2400	Placebo, trastuzumab 8 mg/kg loading dose, followed by the maintenance dose of 6 mg/kg IV every 3 weeks, chemotherapy (anthracycline based or not); n = 2,400	Trastuzumab, pertuzumab, chemotherapy 68.9 (19.4) Placebo, trastuzumab, chemotherapy 69.7 (19.2)	Trastuzumab, pertuzumab, chemotherapy 69.7 (20.0) Placebo, trastuzumab, chemotherapy 71.5 (19.6)	An improvement in HRQoL was shown in the trastuzumab and pertuzumab arm The diarrhea symptoms was more worsening in in the trastuzumab and pertuzumab arm
Sella et al. (2022)	Patients ≥18 years old old Patients has been received surgery for the treatment within 90 days	Trastuzumab 4 mg/kg loading dose followed by 2 mg/kg weekly for 12 weeks, with trastuzumab (6 mg/kg) IV every 21 days for 13 cycles combined with paclitaxel (80 mg/m2); n=82	T-DM1 3.6 mg/kg IV on day1 every 21 days continued for 1 year; n=284	Trastuzumab and paclitaxel -3.87 T-DM1 4.12	Not reported	T-DM1 group has slightly better overall HRQoL score than trastuzumab

**Abbreviations:** ECOG, Eastern Cooperative Oncology Group; IV, intravenous; T-DM1, trastuzumab emtansine.

**TABLE 3:** Profile of HRQoL for cross-sectional studies.

Author, year	Population	Intervention	Comparison	Outcome
**Syrios et al. (2018)**	- Patients >18 and <80 years of age	Subcutaneous trastuzumab 600 mg every 3 weeks, chemotherapy, endocrine therapy; n = 36	Chemotherapy without trastuzumab; n = 27	- Patients in trastuzumab group experienced a better HRQoL
	- Patients had ECOG performance status of 0 or 1			- Patients in trastuzumab groups experienced less diarrhoea, nausea, vomiting, and cognitive and therapy side effects
	- Patients had normal baseline left ventricular ejection fraction			
**Trinca et al (2019)**	- Patients with breast cancer undergoing chemotherapy with or without monoclonal antibodies treatments for at least 2 months	Treatment regimens with trastuzumab, trastuzumab; n = 36	Chemotherapy without trastuzumab; n = 27	- HRQoL was better in trastuzumab group
**Sawaki et al (2022)**	- Patients 70–80 years old	Trastuzumab with chemotherapy; n = 36	Non-trastuzumab group, n = 32	- The overall HRQoL profile among groups was not significantly difference
	- Patients with Eastern Coopertive Oncology Group (ECOG) performance status score 0 or 1			
	- Patients had sufficient organ function	Tratuzumab monotherapy group; n = 52		- Social and family wellbeing domain in trastuzumab with chemotherapy was significantly greater than in trastuzumab monotherapy group
	- Patients who have not received chemotherapy, prior hormonal treatment was allowed			

**Abbreviations:** EORTC-QLQ-C30: European Organization for the Research and Treatment of Cancer Quality of Life Questionnaire; EORTC-QLQ-BR23: The European Organization for Research and Treatment of Cancer Quality of Life Questionnaires-Breast 23.

### 3.6 Interventions

Three studies (Trinca et al., 2019; Sawaki et al., 2020; Taira et al., 2021) compared trastuzumab monotherapy as a frontline treatment in the early stages of Her2-positive breast cancer to trastuzumab combined with chemotherapy. One study (Earl et al., 2020) compared the results of 6 months of trastuzumab with 12 months of trastuzumab. A study (Bines et al., 2021) treated patients with 840 mg loading dose of pertuzumab followed by 420 mg every 3 weeks. All five studies mentioned previously used 8 mg/kg of intravenous trastuzumab as the loading dose, followed by a maintenance dose of 6 mg/kg every 3 weeks. Two studies compared the use of T-DM1 with the use of intravenous trastuzumab at 6 mg/kg combined with adjuvant endocrine (Conte et al., 2020) and paclitaxel 80 mg/m^2^ (Sella et al., 2022). One study (Syrios et al., 2018) compared the use of 600 mg of subcutaneous trastuzumab every 3 weeks combined with chemotherapy and endocrine therapy to a control group using chemotherapy without trastuzumab. Furthermore, an adjuvant trastuzumab-docetaxel-based regimen was used in one study (Au et al., 2013), with a loading dose of 4 mg/kg of trastuzumab.

### 3.7 Health-related quality of life profile

Overall, the patient’s HRQoL score during treatment measured by EORTC-QLQ-C30 favored the trastuzumab group, showing an improvement score from the baseline (Au et al., 2013; Conte et al., 2020). A similar pattern also depicted in a cross-sectional study that patients in the trastuzumab arm experienced a better HRQoL (Syrios et al., 2018; Trinca et al., 2019; Sawaki et al., 2022).

After withdrawing the treatment, the mean change of baseline of HRQoL returned to the baseline levels, showing a better score of HRQoL in the trastuzumab arm (Au et al., 2013; Conte et al., 2020). For physical functioning, the trastuzumab group exhibited a significant improvement from baseline at the mid-point of observations and demonstrated a similar profile after 12months of follow-up (Au et al., 2013; Conte et al., 2020; Bines et al., 2021).

### 3.8 Methodological quality

**FIGURE 2:**
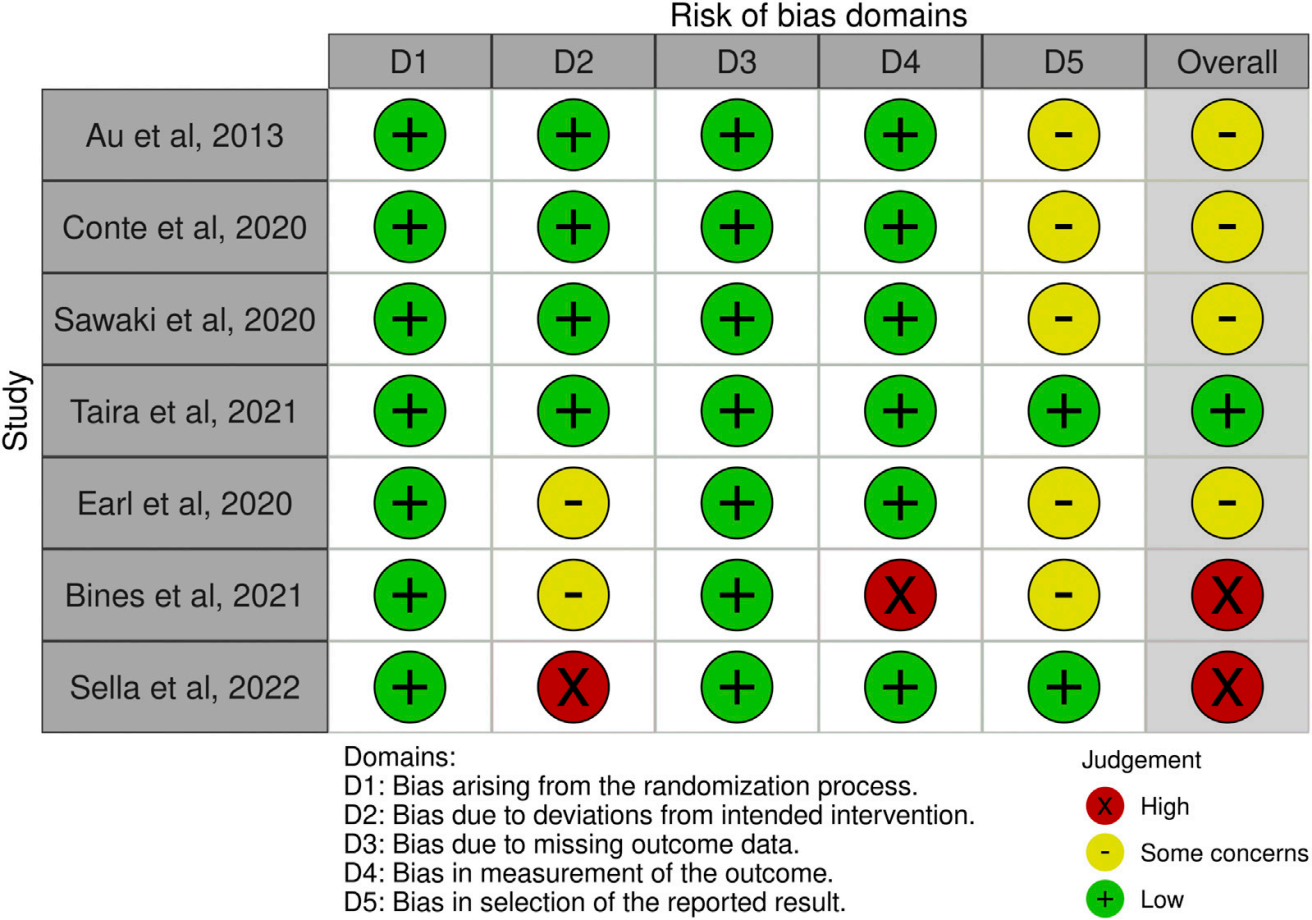
Result of risk of bias using RoB 2.0 tools.

The methodological quality assessment in this systematic review and meta-analysis was conducted according to each article’s study design. The assessment results using RoB 2.0 are summarized in Figure 2. An adequate description of the randomization process was present in all studies. Deviations from the intended interventions were adequately explained in four trials (Au et al., 2013; Conte et al., 2020; Sawaki et al., 2020; Taira et al., 2021). All studies made use of sufficiently large study populations but only two studies (Au et al., 2013; Conte et al., 2020) included an intention-to-treat analysis to overcome the loss of follow-up participants. The outcomes of the trials were assessed appropriately in each of the studies. Only one study insufficiently reported the result which may lead to bias in selection of the reported result (Bines et al., 2021). However, a concern shown in the selection of the reported result for studies (Au et al., 2013; Conte et al., 2020; Earl et al., 2020; Sawaki et al., 2020; Bines et al., 2021) does not present whether the assessed result is likely to have been selected based on the result and from multiple eligible outcome measurements within the outcome domain.

**FIGURE 3:**
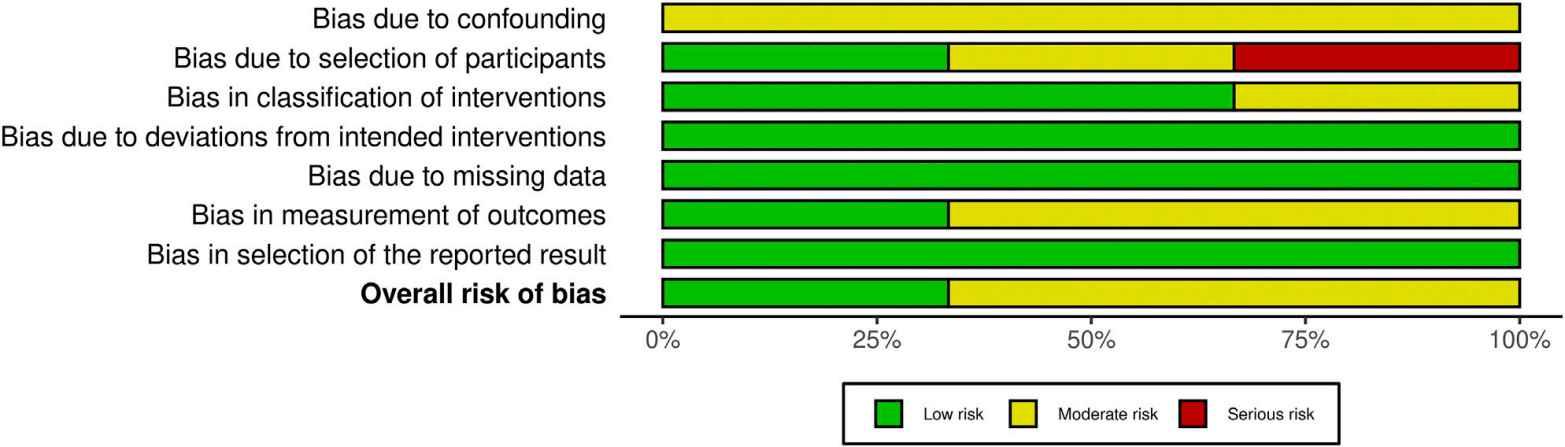
Risk of bias of observational studies using ROBINS-I.

The risk of bias using the ROBINS-I tool is reported in Figure 3. According to the evaluation, the results suggest that the studies quality of reporting was adequate by presenting a low and risk of bias. All confounders in all the studies were measured and controlled, and the reliability and validity of measurement of important domains were sufficient. However, in one study (Sawaki et al., 2022), the selection into the study may have been related to intervention and outcome and there was no information regarding the adjustment techniques used to correct the presence of selection biases. The study failed to explain how to calculate the sample size. In terms of statistical analysis, the study also failed to adequately report on missing data. The quality assessment of the studies also discovered a flaw in the ROBINS-I tool, which lacked a way to address the missing participants in the research and the calculation of the sensitivity analysis. Moreover, the number of outcomes was only shown in the study using a single moment of observation.

### 3.9 Quality of the evidence

There is low-quality of evidence that in RCTs, patients in trastuzumab arm had a better profile of HRQoL than those in chemotherapy group during receiving treatment (MD = 1.92, 95% CI = 1.59 to 2.25, two studies with 2,758 participants). After 12 months of treatment, there is also the low quality of evidence that the HRQoL profile in the trastuzumab arm was comparable with chemotherapy arm (MD = −0.70, 95% CI = −1.62 to 0.22, two studies with 2,111 participants). For observational studies, moderate quality of evidence was presented in showing the higher HRQoL profile in trastuzumab patients compared to the chemotherapy group (MD = 9.29, 95% CI = 1.31 to 17.27, *p* = 0.02, two studies with 108 participants). The result of the quality of the evidence was depicted in Table 4.

**TABLE 4:** Quality of evidence.

Certainty assessment							Number of patients		Effect		Certainty	Importance
Number of studies	Study design	Risk of bias	Inconsistency	Indirectness	Imprecision	Other considerations	[Intervention]	[Comparison]	Relative (95% CI)	Absolute (95% CI)		
The mean change from baseline of HRQoL during treatment between trastuzumab regimen and non-trastuzumab regimen based on RCTs												
2	Randomized trials	Serious	Serious	Not serious	Not serious	None	1,404	1,354	-	MD **1.92 higher** (1.59 higher to 2.25 higher)	⊕⊕○○	IMPORTANT
											Low	
The mean change from baseline of HRQoL after 12 months of follow-up between trastuzumab regimen and non-trastuzumab regimen based on RCTs												
2	Randomized trials	Serious	Serious	Not serious	Not serious	None	1,071	1,040	-	MD **0.7 lower** (1.62 lower to 0.22 higher)	⊕⊕○○	IMPORTANT
											Low	
HRQoL during treatment between trastuzumab regimen and non-trastuzumab regimen chemotherapy based on cross-sectional studies												
2	Observational studies	Not serious	Not serious	Not serious	Serious	None	54	54	-	MD **9.29 higher** (1.32 higher to 17.27 higher)	⊕⊕⊕○	CRITICAL
											Moderate	

### 3.10 Meta-analysis

**FIGURE 4:**
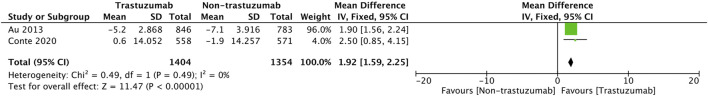
Pooled analysis of the mean change from baseline of HRQoL during treatment based on RCTs.

For RCT studies, a pooled analysis was performed to calculate the pool’s difference in mean change from baseline during treatment in 5 cycles of treatment. A total of 2,758 patients (1,404 patients in the trastuzumab group and 1,354 in the control group) were included in the trials. In Figure 4, it is shown that the mean change from baseline significantly favors the trastuzumab group (MD = 1.92, 95% CI = 1.59 to 2.25, *p* < 0.05) with low heterogeneity (I^2^ = 0%).

**FIGURE 5:**
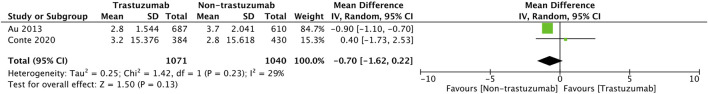
Pooled analysis of the difference in mean change from baseline of HRQoL after 12 months of follow-up based on RCTs.

Another pooled analysis calculated the pool’s difference in mean change from the baseline of HRQOL after 12 months, which is presented in Figure 5. The result shows no significant difference between the two groups of comparison (MD = −0.70, 95% CI = −1.62 to 0.22, *p* = 0.13) with low heterogeneity (I^2^ = 29%).

**FIGURE 6:**
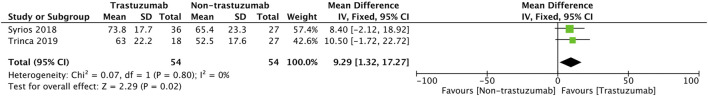
Pooled analysis of HRQoL during treatment based on cross-sectional studies.

For observational studies (Figure 6), the pool’s mean difference in HRQoL between the two comparison groups tended significantly in favor of the trastuzumab regimen (MD = 9.29, 95% CI = 1.31 to 17.27, *p* = 0.02) and a homogenous observation was shown (I^2^ = 0%).

## 4 Discussion

Evaluating patients health status during the early stages of breast cancer is essential in defining the patients perceptions of long-term treatment, treatment benefits and potential adverse impacts. This review focuses on the HRQoL of patients, specifically the changes in HRQoL during and after treatment, to create a comprehensive picture of patients HRQoL when following a regimen including trastuzumab in early disease. The result from this review revealed that in the middle of treatment, the favorable HRQoL profile of patients using EORTC-QLQ C30 in RCT studies (Au et al., 2013; Conte et al., 2020; Earl et al., 2020; Sawaki et al., 2020; Taira et al., 2021) was shown in the trastuzumab arm by demonstrating a better score in the HRQoL, physical, and systemic side effect domains. Patients in trastuzumab arms showed a more tolerable profile of systemic effects domain compared to those in the chemotherapy group and the scores were significantly improved until the end of treatment. A chemotherapy regimen arm showed more deterioration of the mean HRQoL score from baseline until cycle 5 of the treatment, including the cognitive, physical, and fatigue domains. A significant worsening of the nausea/vomiting domain was also observed in the chemotherapy group compared to the trastuzumab group, which included worse nausea, decreased platelet count, and increased liver enzyme. After 12 months of treatment, the HRQoL status had increased in both trastuzumab and chemotherapy groups, presenting a similar pattern but was slightly higher in the trastuzumab group. The deterioration caused by the adverse effects of the treatment seemed resolved, leading to a pattern of increased HRQoL in both groups over time (Conte et al., 2020).

Trastuzumab also demonstrated a beneficial effect for the elderly by providing a clinically meaningful improvement in HRQoL score. For the elderly, HRQoL as an outcome is an essential factor besides a greater chance of survival (Wedding et al., 2007; Muss et al., 2009; Kornblith et al., 2011). Trastuzumab monotherapy exhibited a better score of HRQoL and the combination of trastuzumab with chemotherapy indicated a significant deterioration of patients HRQoL at 2 months and 1 year of treatment.

Furthermore, fewer adverse effects appeared during the first 36 months of observation, and it performed impressively in all functional domains. After 36 months, diminishing adverse effects were observed and HRQoL was unaffected (Taira et al., 2021). Another published study presented a deterioration of the HRQoL during the initial stages of treatment of elderly patients receiving good adjuvant therapy (Cheng et al., 2018). Based on these findings, adding chemotherapy to a trastuzumab regimen leads to adverse effects in older women and a meaningful deterioration of HRQoL (Sawaki et al., 2020). Trastuzumab combined with chemotherapy was related to worse outcome despite favorable cardiotoxicity (Goldvaser et al., 2019).

The above results were supported by the pooled analysis that calculated the mean change from baseline during treatment (Figure 3) and in the follow-up observation (Figure 4) based on two studies (Au et al., 2013; Conte et al., 2020). The pooled result showed that patients in the trastuzumab group experienced a meaningfully better HRQoL during treatment, and deterioration was more prevalent in the chemotherapy group. The lower mean improvement from baseline in HRQoL in the chemotherapy group was associated with the adverse events caused by chemotherapy. Breast cancer patients undergoing chemotherapy demonstrate less favorable score of HRQoL due to increasing symptoms attributed to systemic treatment (Leinert et al., 2017; Binotto et al., 2020). A cohort study also demonstrated a worsening score of HRQoL and functional scales while there was a meaningful increase in the domain of fatigue, nausea and vomiting, insomnia, appetite loss and diarrhea (Binotto et al., 2020). These findings are consistent with the APHINITY trial (Bines et al., 2021) investigating the HRQoL of patients in the early stages of breast cancer, which, using EORTC-QLQ-BR30, revealed that patients experienced a deterioration of HRQoL and functioning domain while receiving taxane-based chemotherapy. The patients using anthracycline-cyclophosphamide-based chemotherapy and docetaxel experienced a lower score in general health, physical functioning, and systemic side effects during treatment (Au et al., 2013) due to cardiotoxicity, fatigue, and nausea (Mills et al., 2005; Cohen et al., 2007; Cardinale et al., 2020).

For cross-sectional studies (Syrios et al., 2018; Trinca et al., 2019; Sawaki et al., 2022), patients had higher scores of HRQoL in the trastuzumab group compared to the chemotherapy group. Patients receiving trastuzumab had a better HRQoL score than the chemotherapy group, and more patients experienced decreased nausea/vomiting, cognitive side effects, and systemic therapy side effects (Syrios et al., 2018). However, patients who followed a chemotherapy regimen combined with intravenous trastuzumab had more diarrhea (Trinca et al., 2019). Another meta-analysis (J, 2019) also revealed a higher risk of diarrhea during trastuzumab therapy. That said, another study assessing subcutaneous trastuzumab demonstrated less diarrhea, as well as a significantly lower score in nausea/vomiting (Syrios et al., 2018). Generally, patients undergoing subcutaneous trastuzumab had a similar HRQoL profile to those receiving intravenous administration (Syrios et al., 2018). Subcutaneous trastuzumab has comparable efficacy, safety, and profile and requires a shorter period of administration than intravenous trastuzumab (G et al., 2012; Shpilberg and Jackisch, 2013; Schmidt et al., 2021). The PrefHer trial (Pivot et al., 2014) mentioned that most patients (88.9%) preferred SC administration due to shorter administration, less pain, and fewer side effects. The patients receiving subcutaneous trastuzumab during the early stages of breast cancer experienced less severe symptoms and a general improvement in functioning. The pooled analysis (Figure 5) also supports this result, as the pooled difference of the HRQoL between the two studies trended meaningfully towards the regimen including trastuzumab (Syrios et al., 2018; Trinca et al., 2019).

When evaluating treatment benefits, it is essential to consider the effect on HRQoL of adding a new drug to a treatment regimen (Johansson et al., 2008; Osoba et al., 2016). Ideally, the addition should improve treatment efficacy without causing deterioration in the HRQoL. This review shows that adding trastuzumab to chemotherapy supports efficacy and doesn’t lead to increased deterioration of the patient’s HRQoL. The improvement in HRQoL is likely associated with a decrease in tumor mass (Osoba et al., 2002). According to an efficacy study (Slamon et al., 2001), a high proportion of patients treated with trastuzumab and chemotherapy showed a reduction in tumors that contributed to a longer time of disease progression and the survival was longer compared to those undergoing chemotherapy alone. Several RCTs (Gianni et al., 2011; Perez et al., 2011; Slamon et al., 2011) and a large cohort study (Bonifazi et al., 2014) evaluating the efficacy of trastuzumab in the Her2-positive early breast cancer management exhibited that the addition of trastuzumab in the chemotherapy regimen was associated in the longer survival. Despite a comparable HRQoL profile between trastuzumab and chemotherapy 1 year after treatment, the benefit of the trastuzumab in the HRQoL of patients potentially supports the overall survival due to the favorable score of the mean change of baseline during treatment in trastuzumab patients. A deterioration of HRQoL leads to significantly related to shorter survival (van Nieuwenhuizen et al., 2021). In addition, the improved chances of survival as a result of trastuzumab should balance out the impact on patients HRQoL. Another published article found that a better score of HRQoL was meaningfully associated with an improvement of 5-year survival (van Nieuwenhuizen et al., 2021). Additionally, this result of this review may answer the limited used of trastuzumab in low- and middle-income countries (LMIC) due to its high cost.

To the best of our knowledge, this is the first systematic review and meta-analysis of the effect of trastuzumab in the early stages of breast cancer management on HRQoL. Three large electronic databases without year and language limitations were used to conduct a systematic search and we applied strict inclusion criteria to the articles we found. The RoB 2.0. ROBINS-I, and GRADE were used to comprehensively assess the risk of bias and the level of evidence of the studied included in this review. In addition, we deemed the studies of sufficient quality despite some of the cross-sectional studies lacking certain information. This review may provide a comprehensive assessment of the available evidence on this topic, including studies that have been published up until the present day, that can potentially inform and enhance use in settings where trastuzumab is not yet used large scale in all countries, such as a low-middle income country like Indonesia with numerous remote areas. However, there were several limitations to this review. The variety of treatment regimens in the trastuzumab and control groups may affect the different HRQoL profiles and result in high risk of bias which reduce the quality of the evidence. However, in the synthesis process, the heterogeneity was still possible to reduce from another viewpoint as the studies have the same HRQoL instrument, further enhancing the relevance of this analysis. In this case, we suggest that future studies in this area should consider using consistent comparator arms. Of the overall results of the four articles presented in the meta-analysis in this study, two lent themselves less clearly to interpretation, despite the high level of homogeneity in the calculation. The meta-analysis result was supported by the GRADE, in which shows low to moderate quality of evidence.

## 5 Conclusion

 Trastuzumab as a targeted therapy may result in an improvement of HRQoL in the early stages of Her2-positive breast cancer. The measurement from RCTs revealed a more favorable score of HRQoL and a tolerable profile in the systemic side effects domain. A meaningfully better score of HRQoL in cross-sectional studies also provided a beneficial effect of trastuzumab in the management of Her-2 positive early breast cancer. These findings of significant improvements in patients HRQoL and less clinically significant deterioration in side effects of trastuzumab-containing regimen during treatment were supported by prolonged survival.

## Data Availability

The original contributions presented in the study are included in the article/Supplementary Material, further inquiries can be directed to the corresponding author.
