# Supplementary material for: Health-related quality of life in Her2-positive early breast cancer woman using trastuzumab: A systematic review and meta-analysis
Source: Front Pharmacol. 2023 Apr 14;14:1090326. doi: 10.3389/fphar.2023.1090326 (PMC10140570; doi:10.3389/fphar.2023.1090326)
Supplement: Supplementary file 1 [file Table1.DOCX]

Supplementary Material

# Supplementary Tables

**Table A. Search Strategy and Results**

| No | Database | Search code | Search terms | Hits |
| --- | --- | --- | --- | --- |
| 1 | PubMed | #1 | "Breast Neoplasms"[MeSH Terms] OR ("breast"[Title/Abstract] OR "mamma"[Title/Abstract] OR "mammary"[Title/Abstract]) | 597334 |
|  |  | #2 | ("carcinoma*"[Title/Abstract] OR "tumor*"[Title/Abstract] OR "tumour*"[Title/Abstract] OR "neoplasm*"[Title/Abstract] OR "cancer*"[Title/Abstract] OR "malignan*"[Title/Abstract]))) | 3877357 |
|  |  | #3 | "Trastuzumab"[MeSH Terms] OR "Trastuzumab"[Title/Abstract] OR "herceptin"[Title/Abstract] | 14528 |
|  |  | #4 | "Quality of Life"[MeSH Terms] OR "Quality-Adjusted Life Years"[MeSH Terms] OR "Quality of Life"[Title/Abstract] OR "HRQOL"[Title/Abstract] OR "HRQL"[Title/Abstract] OR "health-related quality of life"[Title/Abstract] OR "health related quality of life"[Title/Abstract] OR "health quality of life"[Title/Abstract] OR "life quality"[Title/Abstract] OR "QOL"[Title/Abstract] OR "quality-adjusted life year*"[Title/Abstract] OR "quality adjusted life year*"[Title/Abstract] OR "qaly*"[Title/Abstract] OR "healthy years equivalent*"[Title/Abstract] OR "adjusted life year*"[Title/Abstract] OR "utilit*"[Title/Abstract] | 688242 |
|  |  |  | #1 AND #2 AND #3 AND #4 | 563 |
| 2 | Embase (Ovid®) | #1 | 'breast tumor'/exp OR 'breast tumor' OR ('breast':ab,ti OR 'mamma':ab,ti OR 'mammary':ab,ti) | 883673 |
|  |  | #2 | 'carcinoma*':ab,ti OR 'tumor*':ab,ti OR 'tumour*':ab,ti OR 'neoplasm*':ab,ti OR 'cancer*':ab,ti OR 'malignan*':ab,ti | 5221435 |
|  |  | #3 | 'trastuzumab'/exp OR 'trastuzumab':ab,ti OR 'herceptin':ab,ti | 49641 |
|  |  | #4 | 'quality of life'/exp OR 'quality-adjusted life years'/exp OR 'quality of life':ab,ti OR 'hrqol':ab,ti OR 'hrql':ab,ti OR 'health-related quality of life':ab,ti OR 'health related quality of life':ab,ti OR 'health quality of life':ab,ti OR 'life quality':ab,ti OR 'qol':ab,ti OR 'quality-adjusted life year*':ab,ti OR 'quality adjusted life year*':ab,ti OR 'qaly*':ab,ti OR 'healthy years equivalent*':ab,ti OR 'adjusted life year*':ab,ti OR 'utilit*':ab,ti | 1095338 |
|  |  |  | #1 AND #2 AND #3 AND #4 | 2753 |
| 3 | Scopus | #1 | "Breast Neoplasms"  OR  ( ( "breast"  OR  "mamma"  OR  "mammary" ) ) | 908805 |
|  |  | #2 | "carcinoma*" OR "tumor*" OR "tumour*" OR "neoplasm*" OR "cancer*" OR "malignan*" | 6016662 |
|  |  | #3 | "Trastuzumab" OR "Trastuzumab" OR "herceptin" | 38390 |
|  |  | #4 | "Quality of Life" OR "Quality-Adjusted Life Years" OR "Quality of Life" OR "HRQOL" OR "HRQL" OR "health-related quality of life" OR "health related quality of life" OR "health quality of life" OR "life quality" OR "QOL" OR "quality-adjusted life year*" OR "quality adjusted life year*" OR "qaly*" OR "healthy years equivalent*" OR "adjusted life year*" OR "utilit*" | 1338455 |
|  |  |  | #1 AND #2 AND #3 AND #4 | 2436 |

**Note: The search until February 2023**

**Table B. Risk of bias assessment for RCTs using RoB 2 tool**

| **Basic information** | **Domain 1. Randomization process** | | | | | **Domain 2. Deviations from intended interventions** | | | | | | | | | | | | **Domain 3. Mising outcome data** | | | | | | | | | | | |  |
| --- | --- | --- | --- | --- | --- | --- | --- | --- | --- | --- | --- | --- | --- | --- | --- | --- | --- | --- | --- | --- | --- | --- | --- | --- | --- | --- | --- | --- | --- | --- |
| **Study ID** | **1.1** | **1.2** | **1.3** | **1.0 Algorithm result** | **1.0 Assessor's Judgement** | **2.1** | **2.2** | **2.3** | **2.4** | **2.5** | **2.6** | | **2.7** | **2.0 Algorithm result** | | **2.0 Assessor's Judgement** | | **3.1** | | **3.2** | | **3.3** | | **3.4** | | **3.0 Algorithm result** | | **3.0 Assessor's judgement** | |  |
| Au et al, 2013 | Y | PY | PN | Low | Low | N | PN | Y | PN | NA | NA |  | | | Low | | Low | | Y | | NA | | NA | | NA | | Low | | Low | |
| Conte et al, 2020 | Y | Y | N | Low | Low | N | PY | Y | PN | NA | NA |  | | | Low | | Low | | PN | | Y | | NA | | NA | | Low | | Low | |
| Sawaki et al, 2020 | Y | Y | N | Low | Low | N | PN | NA | PN | NA | NA |  | | | Low | | Low | | Y | | NA | | NA | | NA | | Low | | Low | |
| Taira et al, 2021 | Y | PY | N | Low | Low | NI | PY | Y | N | NA | NA |  | | | Low | | Low | | Y | | NA | | NA | | NA | | Low | | Low | |
| Earl et al, 2020 | Y | Y | N | Low | Low | Y | Y | NI | PN | NA | Y |  | | | Some concerns | | Some concerns | | Y | | NA | | NA | | NA | | Low | | Low | |
| Bines et al, 2021 | Y | PY | N | Low | Low | NI | PN | PN | NA | NA | NI | PN | | | High | | Some concerns | | Y | | NA | | NA | | NA | | Low | | Low | |
| Sella et al, 2022 | Y | Y | N | Low | Low | NI | NI | NI | NA | NA | N | NI | | | High | | High | | PY | | NA | | NA | | NA | | Low | | Low | |

*Cont…* **Table B. Risk of bias assessment for RCTs using RoB 2 tool**

| **Basic information** | **Domain 4. Measurement of the outcome** | **Domain 5. Selection of the reported result** | | | | | **Domain 6. Overall Bias** | |
| --- | --- | --- | --- | --- | --- | --- | --- | --- |
| **Study ID** | **4.0 Note for optional question** | **5.1** | **5.2** | **5.3** | **5.0 Algorithm result** | **5.0 Assessor's Judgement** | **Algorithm's overall Judgement** | **Assessor's overall Judgement** |
| Au et al, 2013 |  | NI | PN | N | Some concerns | Some concerns | Some concerns | Some concerns |
| Conte et al, 2020 |  | NI | N | N | Some concerns | Some concerns | Some concerns | Some concerns |
| Sawaki et al, 2020 |  | NI | N | N | Some concerns | Some concerns | Some concerns | Some concerns |
| Taira et al, 2021 |  | Y | N | PN | Low | Low | Low | Low |
| Earl et al, 2020 |  | NI | N | N | Some concerns | Some concerns | Some concerns | Some concerns |
| Bines et al, 2021 |  | PN | PN | PN | Some concerns | Some concerns | High | High |
| Sella et al, 2022 |  | Y | N | N | Low | Low | High | High |

Note: Y=Yes; PY=Probably Yes; N: No; PN: Probably No; NI: No Information; NA=Not Applicable

Each domain received an overall risk of bias based on decision tree provided by the RoB 2 assessment

**Table B. Risk of bias assessment for cross-sectional studies using ROBINS-I**

| Articles | | Trinca et al, 2019 | Syrios et al, 2018 | Sawaki et al, 2022 |
| --- | --- | --- | --- | --- |
| Bias due to confounding | 1.1 | PN | N | N |
|  | 1.2 | N | N | N |
|  | 1.3 | / | / | / |
|  | 1.4 | NI | NI | NI |
|  | 1.5 | NI | NI | NI |
|  | 1.6 | N | N | N |
|  | 1.7 | NI | NI | NI |
|  | 1.8 | NI | NI | NI |
|  | Risk of bias judgement | Moderate | Moderate | Moderate |
| Bias in selection of particiants into the study | 2.1 | PN | PY | Y |
|  | 2.2 | PN | PY | Y |
|  | 2.3 | PY | PY | PY |
|  | 2.4 | PY | PN | PY |
|  | 2.5 | N | NI | PY |
|  | Risk of bias judgement | Low | Moderate | Serious |
| Bias in classification of interventions | 3.1 | Y | Y | Y |
|  | 3.2 | Y | Y | Y |
|  | 3.3 | PN | PN | Y |
|  | Risk of bias judgement | Low | Low | Moderate |
| Bias due to the deviations from intended interventions | 4.1 | N | N | N |
|  | 4.2 | N | / | / |
|  | 4.3 | / | / | / |
|  | 4.4 | / | / | / |
|  | 4.5 | / | / | / |
|  | 4.6 | / | / | / |
|  | Risk of bias judgement | Low | Low | Low |
| Bias due to missing data | 5.1 | Y | Y | Y |
|  | 5.2 | N | N | N |
|  | 5.3 | N | N | N |
|  | 5.4 | / | / | / |
|  | 5.5 | / | / | / |
|  | Risk of bias judgement | Low | Low | Low |
| Bias in measurement of outcomes | 6.1 | NI | PY | PY |
|  | 6.2 | NI | PY | PY |
|  | 6.3 | PY | PY | PY |
|  | 6.4 | PN | PN | PN |
|  | Risk of bias judgement | Low | Moderate | Moderate |
| Bias in selection of the reported result | 7.1 | N | N | N |
|  | 7.2 | N | N | N |
|  | 7.3 | N | N | N |
|  | Risk of bias judgement | Low | Low | Low |
| Overall bias |  | Low | Moderate | Moderate |

Note: Y=Yes; PY=Probably Yes; N: No; PN: Probably No; NI: No Information

Each domain received an overall risk of bias based on decision tree provided by the ROBINS-assessment
